# HOTAIRM1 competed endogenously with miR‐148a to regulate DLGAP1 in head and neck tumor cells

**DOI:** 10.1002/cam4.1523

**Published:** 2018-06-14

**Authors:** Mei Zheng, Xingguang Liu, Qin Zhou, Gangli Liu

**Affiliations:** ^1^ Department of Traditional Chinese Medicine Qianfoshan Hospital Affiliated to Shandong University Ji'nan 250014 Shandong China; ^2^ Department of Oral and Maxillofacial Surgery School and Hospital of Stomatology Shandong University Ji'nan 250012 Shandong China

**Keywords:** *DLGAP1*, head and neck tumors, HOTAIRM1, miR‐148a

## Abstract

This study is aimed to explore the regulatory effect of lncRNA HOTAIR/miR‐148a/*DLGAP1* axis on head and neck tumor (HNT) cell growth, cell mobility, and invasiveness. HOTAIRM1, miR‐148a, and *DLGAP1* level in HNT tissues and adjacent normal tissues were measured by qRT‐PCR. Cell Counting Kit‐8 (CCK‐8) and Transwell (migration and invasion) assay were used to survey the influence of HOTAIRM1, miR‐148a, and *DLGAP1* on Fadu cells. Nude mouse xenograft was utilized to validate the influence of HOTAIRM1 in vivo. Dual‐luciferase reporter assay confirms the relationship between HOTAIRM1 and miR‐148a, miR‐148a, and *DLGAP1*. The expression level of HOTAIRM1 was downregulated in human HNT tissues and cells. Overexpression of HOTAIRM1 significantly moderated Fadu cells proliferation, apoptosis, migration, and invasion in vitro and impaired the tumorigenesis in vivo. The expression level of miR‐148a was upregulated in human HNT tissue compared to the adjacent tissues. We identified that miR‐148a was a target of HOTAIRM1 and its expression levels were reduced by HOTAIRM1. Transfection of miR‐148a mimics increased proliferation, migration, and invasion of Fadu cells. *DLGAP1* was identified as a novel target of miR‐148a and its expression level was promoted by either HOTAIRM1 overexpression or miR‐148a knockdown. Overexpression of *DLGAP1* also facilitated the cell viability and metastasis of Fadu cells. HOTAIRM1 was confirmed as a tumor suppressor via sponging miR‐148a and promote the expression of *DLGAP1*, which could be regarded as an important target for the prevention and treatment of HNT.

## Introduction

Head and neck tumor (HNT) is one of the most common diagnosed malignancies worldwide, with over 600,000 new cases have reported each year 1. And the reported five‐year mortality rate was approximately 50% despite considerable advances in treatment and increased knowledge about therapy 2. Tobacco smoking, alcohol consumption, and human papilloma virus (HPV) infections have been reported to be associated with the occurrence of HNT 3. Frequent recurrences and distant metastasis of cancer were believed to be the main factors resulting in poor efficacy for HNT therapy 4. Thus, to develop effective treatment against HNT, understanding pathological metastasis or progression of HNT is urgently required 5.

HOX antisense intergenic RNA myeloid 1 (HOTAIRM1), a long noncoding RNA (lncRNA), was found recently to participate in various tumor metastasis 6, 7. HOTAIRM1 may act as regulator of gene expression during myelopoiesis which is expressed from *HOXA* genomic cluster between *HOXA1* and *HOXA2* 8. HOTAIRM1 has been demonstrated to play a role in various cancers, including colorectal cancer 9, adenocarcinoma 10, breast cancer 11, and acute myeloid leukemia 7. Furthermore, HOTAIRM1 was also detected in fetal brain, with the implication that it had a new function in HNT 9. However, whether HOTAIRM1 contributes to the malignant progression of HNT remains unclear. To explore the role of HOTAIRM1 in HNT, we manipulated its level in Fadu cells, detected the variation of cell biological functions, and investigated the underlying molecular pathways.

MicroRNA (miRNA, 18–22 nt), the endogenous short noncoding single‐stranded RNA, could directly induce messenger RNA (mRNA) degradation or inhibit translation by interacting with the 3′‐UTR of target mRNAs 12. MicroRNAs are involved in many cellular physiological processes via complex mechanisms. MicroRNA‐148a (miR‐148a) is aberrantly expressed in various cancers and has been characterized for an oncogenic or tumor suppressor depending on the specific targets 13. For instance, Ying et al. reported that miR‐148a was significantly upregulated during laryngeal squamous cell carcinoma carcinogenesis 14. Kim et al. 15 verified that upregulated miR‐148a could accelerate the malignant process in glioblastoma cell through targeting *MIG6* and *BIM*, and miR‐148a was significantly upregulated in samples of 89 osteosarcoma patients which was reported by Ma et al. 16. Whereas, miR‐148a has also been reported to serve as a tumor inhibitor in majority cancers, including gastric cancer 17, colorectal cancer 18, and oral cancer 19. Despite the numerous studies, researches on the interaction of miR‐148a with targeting genes in HNT are still discrepancy. *DLGAP1*, as a targeting gene of miR‐148a predicted by bioinformatics analysis, is known to link with morphology and behavior of the brain cell, particularly in the nervous system 20. However, the concrete mechanism by which *DLGAP1* exerts its role in HNT, especially its interaction with noncoding RNA such as miRNAs and lncRNAs, remains obscure.

The purpose of our study was to investigate the function and the underlying mechanism of lncRNA HOTAIRM1 in HNT. The influence of HOTAIRM1 on HNT cells was studied in vitro and in vivo. We evaluated the expression of HOTAIRM1 and its correlation with clinicopathologic features in HNT patients. We also explored the role of HOTAIRM1/miR‐148a/*DLGAP1* axis in HNT.

## Materials and Methods

### Patients and tissue samples

HNT tissues were obtained from 109 patients who were admitted to the School and Hospital of Stomatology, Shandong University. None of the patients received radical treatment or chemotherapy before surgical treatment. All sample tissues were frozen in liquid nitrogen and then stored at −80°C. All human specimens were obtained with the approval by the Institutional Ethics Committee of School and Hospital of Stomatology, Shandong University, and all samples were supplied by patients who provided informed consent.

### Cell lines and cultures

Human nasopharyngeal epithelial cells (NP69 cell), human tongue squamous carcinoma cells (Tca‐8113 cell), human laryngeal squamous carcinoma cells (TU177 cell), human nasopharyngeal carcinoma cells (HNE1 cell), and human hypopharyngeal tumor cells (Fadu cell) were purchased from BeNa Culture Collection (Beijing, China). NP69 cell, Tca‐8113 cell, and HNE1 cell were cultivated in RPMI‐1640 medium supplemented with 10% fetal bovine serum (FBS); TU177 cell were cultivated in high glucose DMEM medium supplemented with 10% FBS; Fadu cells were cultured in MEM medium supplemented with 10% fetal bovine serum (FBS), 100 *μ*/mL penicillin and 100 mg/mL streptomycin. All cells were cultured under humidified atmosphere with 5% CO_2_.

### Cell transfection

PcDNA3.1‐HOTAIRM1, pcDNA3.1‐DLGAP1, pcDNA3.1 plasmid vectors, siRNA1‐HOTAIRM1, siRNA2‐HOTAIRM1, miR‐148a mimics, mimics control, miR‐148a inhibitor, and inhibitor control were commercially obtained from GenePharma (Shanghai, China). The sequence of HOTAIRM1 siRNA listed in Table 1. The Fadu cells were plated in 6‐well plates at a density of 1 × 10^6^ per well and cultured in incubator for 24 h at 37°C in 5% CO_2_ until the cell confluence arrived 80–90%. Transfections were executed by Lipofectamine 2000 (Invitrogen, Carlsbad, CA) following the instructions of manufacturer. The medium was replaced with complete medium after 6‐hour transfection.

**Table 1:** siRNA sequence of HOTAIRM1

Gene	Sequence
siRNA1	5′‐GCCAGAAACCAGCCATAGT‐3′
siNC‐1	5′‐GCCCAAACGACTACGAAGT‐3′
siRNA2	5′‐GGAGCAAACCTATGAAGAA‐3′
siNC‐2	5′‐GGACAAATATCAAGCGGAA‐3′

### RNA isolation and quantitative real‐time PCR (qRT‐PCR)

Total RNA was carried out from the tissues and cells using TRIzol^™^ (Invitrogen), according to the manufacturer's instructions. A PrimeScript^®^1st Strand Synthesis Kit (TaKaRa, Tokyo, Japan) was used to convert RNA (2 *μ*g) into cDNA. Subsequently, qRT‐PCR was performed using QuantiTect SYBR^®^ Green RT‐PCR Kit (QIAGEN, Dusseldorf, Germany) according to the manufacturer's instructions. Relative expression values were calculated using the 2^−ΔΔCt^ method. GAPDH and U6 was utilized as the intern controls. The specific primers used are presented in Table 2.

**Table 2:** qRT‐PCR Primer Sequences

Primer	Accession number	Sequence (5′–3′)
HOTAIRM1	NR_038366.1	
Forward primer		CCCACCGTTCAATGAAAG
Reverse primer		GTTTCAAACACCCACATTTC
miR‐148a	NR_029597.1	
Forward primer		TCAGTGCACTACAGAACTTTGT
Reverse primer		GCTGTCAACGATACGCTACGT
DLGAP1	NM_001242761.1	
Forward primer		TCTCTCGAGTCCTTCCCGTC
Reverse primer		TTCCTTGCTTCCGAGTCAGG
GAPDH	NM_002046.6	
Forward primer		AGCCACATCGCTCAGACAC
Reverse primer		GCCCAATACGACCAAATCC
U6	NR_004394.1	
Forward primer		ATTGGAACGATACAGAGAAGATT
Reverse primer		GGAACGCTTCACGAATTTG

### Western blot

Total protein was extracted using the RIPA buffer (Pierce, Rockford, IL) in the presence of a Protease Inhibitor Cocktail (Pierce). After quantified by the bicinchoninic acid method (Waltham, MA), 80 *μ*g protein was separated by sodium dodecyl sulfate‐polyacrylamide gel electrophoresis (SDS‐PAGE) using the Novex NuPAGE system (Invitrogen) and denatured for 5 min. Subsequently, the protein was transferred to 0.45‐Am nitrocellulose membranes. Membranes were blocked in TBS‐T buffer containing 5% nonfat milk for 1 h and incubated overnight with the following primary antibodies rabbit anti‐human DLGAP1 and GAPDH (1:800; Abcam, Cambridge, MA). After washed using TBST, the membranes were then hybridized with the horseradish peroxidase (HRP)‐linked antibody goat anti‐rabbit IgG (1:2000; Abcam) for 1 h. Signal detection was carried out with an ECL system (Amersham Pharmacia, Piscataway, NJ).

### Cell counting kit‐8

Cell Counting Kit‐8 kit (CCK‐8) (Dojindo, Kumamoto, Japan) was used to detect the effect of the expression of miR‐148a and HOTAIRM1 on cell proliferation. Briefly, cells at a concentration of 2 × 10^3^ per well were seeded in the 96‐well plate and incubated for 24 h, 48 h, 72 h, 96 h, respectively. At the indicated time point, 10 *μ*L CCK‐8 was added, and the plate was incubated for another 4 h. SpectraMax M5 microplate reader (Molecular Devices, Sunnyvale, CA) was applied to measure the absorbance of 450 nm.

### Plate colony formation assay

Briefly, after 24 h of transfection, Fadu cells were initially seeded into 3.5 cm culture dishes at a density of 800 cells per dish and maintained in medium containing 10% FBS, which was refreshed every two days. After the cells had incubated for approximately 2 weeks at 37°C in 5% CO_2_, their colonies were visible to the naked eye. The cells were fixed with 4% paraformaldehyde for 15 min and staining with 0.1% crystal violet for 15 min before being counted. The colony numbers were counted using ImageJ software and manually counted from three randomly chosen fields. Experiments were tested in triplicate.

### Cell apoptosis analysis

A fluorescein‐conjugated Annexin V (Annexin V‐FITC)/propidium iodide (PI) staining kit (BD Biosciences, San Jose, CA) was applied to detect the apoptosis of the cells, following to instructions of the manufacturer's. An FACS Calibur FCM (BD Biosciences) was used to observe cell apoptosis. Experiments in triplicate helped to reduce errors. FACS Diva software was adopted at data analysis.

### Dual‐luciferase activity assay

pmirGLO, pmirGLO‐HOTAIRM1‐wt or pmirGLO‐HOTAIRM1‐mut, pmirGLO‐*DLGAP1*‐wt, or pmirGLO‐*DLGAP1*‐mut were commercially obtained from Genepharma. PmirGLO, pmirGLO‐HOTAIRM1‐wt or pmirGLO‐HOTAIRM1‐mut was cotransfected with miRNA‐148a mimics or mimics control into Fadu cells by Lipofectamine‐mediated gene transfer. pmirGLO, pmirGLO‐*DLGAP1*‐wt or pmirGLO‐*DLGAP1*‐mut was cotransfected with miR‐148a mimics, miR‐148a inhibitor, pcDNA3.1‐HOTAIRM1, p‐HOTAIRM1 + miR‐148a mimics, or p‐HOTAIRM1 + miR‐148a inhibitor into Fadu cells by Lipofectamine‐mediated gene transfer. Cells were harvested 48 h after transfection, and luciferase activity was measured as chemiluminescence in a luminometer (PerkinElmer Life Sciences, Boston, MA) using the dual‐luciferase reporter assay system (Promega, Madison WI) according to the manufacturer's protocol.

### Cell invasion and migration assay

For transwell migration assays, Fadu cells transfected cells (4 × 10^5^) were plated in the top chamber with the noncoated membrane (24‐well insert; pore size, 8 *μ*m; BD Biosciences). For invasion assays, Matrigel (BD biosciences) was polymerized in transwell inserts for 45 min at 37°C. In both assays, cells were plated in the top chamber in medium without serum; the lower chamber was filled with 20% FBS (GIBCO BRL, Grand Island, NY) as a chemoattractant. Cells were incubated for 24 h, and the cells that did not migrate or invade through the pores were removed by a cotton swab. Cells migrated on the lower surface of the membrane were fixed and stained with 0.1% Crystal violet staining solution. The cells on the bottom of the membrane were counted from five different microscopic fields, and the average number was calculated.

### Nude mice allogeneic experiments

Twelve 6‐week‐old nude mice were purchased from Experimental Animal Center at the West China School of Medicine, Sichuan University (Sichuan, China), and were randomly divided into two groups. The serum‐free cell suspensions (1 × 10^7^/mL) of Fadu cells transfected with pcDNA3.1‐HOTAIRM1 or pcDNA3.1‐vector (as negative control) were injected subcutaneously on the back of each mouse (0.2 mL). When the tumor grown to about 100–200 mm^3^, tumor volume was calculated it by the following formula: 1/2 × L^2 ^× W, where L is the length (mm), and W is the width (mm) of the tumor. The average volume of tumor was measured for 3 times every 3 days. At the termination of the experiment (the 21st day), mice were killed, and the tumor was excised from each mouse to measure the average volume and weight. Total RNA was isolated and the expression level of HOTAIRM1 was detected with qRT‐PCR.

### Statistical analysis

Statistical analyses were performed using GraphPad Prism 6.0 (GraphPad Software, La Jolla, CA, USA). All data are presented as the mean ± standard deviation, and the in vitro experiments were performed in triplicate. Differences between two groups were determined by Student's t‐test, and one‐way ANOVA was used for comparison among three groups or more. *P*‐values of <0.05 were considered as a statistically significant difference.

## Results

### Expression of HOTAIRM1 in HNT tissues

**Figure 1:**
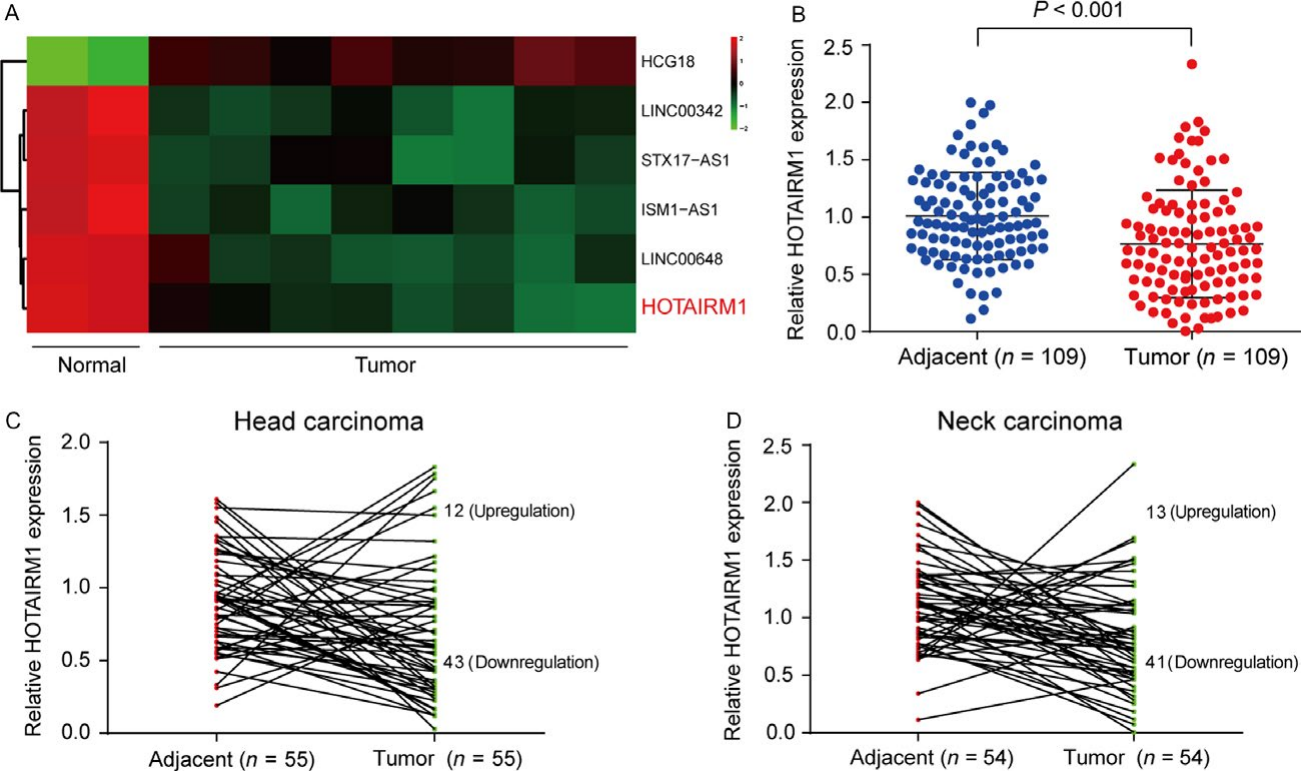
LncRNA microarray analysis and downregulated expression of HOTAIRM1 in HNT tissues (A) The heat map illustrated the relative expression levels of the six lncRNA in tumor and normal tissues. (B) qRT‐PCR was performed to examine the relative expression of HOTAIRM1 in HNT tissues (Tumor) and their matched adjacent tissues (Adjacent). (C) The expression level of HOTAIRM1 in 55 Head carcinoma tissues and its corresponding adjacent normal tissues. (D) The expression level of HOTAIRM1 in 54 Neck carcinoma tissues and its corresponding adjacent tissues. Downregulation, HOTAIRM1 expression decreased in head and neck tumors tissues compared to the adjacent tissues; Upregulation, HOTAIRM1 expression increased in head and neck tumors tissues compared to the adjacent tissues.

Abnormally expressed lncRNAs were identified by microarray analysis in HNT tissues compared with adjacent tissues. By analyzing the GSE21644 chip based on the Affymetrix Human Genome U133 Plus 2.0 Array platform, five significantly downregulated lncRNAs were found under the condition of false positive rate <0.05 and Fold Change >2, and the expression of HOTAIRM1 was the lowest (Fig. 1A). We next examined the HOTAIRM1 expression in 109 HNT patient samples and confirmed the downregulation of HOTAIRM1 in tumor tissues than that in adjacent tissues (Fig. 1B). According to the tumor location, patients were divided into two groups as Head carcinoma and Neck carcinoma. The expression level of HOTAIRM1 in 43 Head carcinoma tissues (55 in total) (Fig. 1C) and 41 Neck carcinoma tissues (54 in total) (Fig. 1D) was downregulated compared to the corresponding adjacent normal tissues. The correlation between HOTAIRM1 expression and clinicopathological features in HNT was listed in Table 3. There was no significant association between HOTAIRM1 level and age, gender, or tumor location, while patients with high expression of HOTAIRM1 are more potential to get an advanced TNM stage (*P *<* *0.01).

**Table 3:** The relationship between HOTAIRM1 expression and clinicopathological parameters in head and neck tumor

Clinicopathological parameters	Number	HOTAIRM1 expression level (T/N)^a^		*P*
		Average value	SD	
Age				
≤60	49	1.35	0.87	0.2368
>60	60	1.17	0.71	
Gender				
Male	50	1.24	0.66	0.8974
Female	59	1.26	0.91	
Tumor location				
Head	55	1.32	0.82	0.3460
Neck	54	1.18	0.72	
TNM staging				
Early stage (I‐II)	65	1.50	1.01	0.0069
Advanced (III–IV)	44	1.03	0.77	

a The relative level of HOTAIRM1 is the ratio of tumor tissue to normal tissue.

### Effects of HOTAIRM1 on Fadu cells proliferation, apoptosis, migration, and invasion

**Figure 2:**
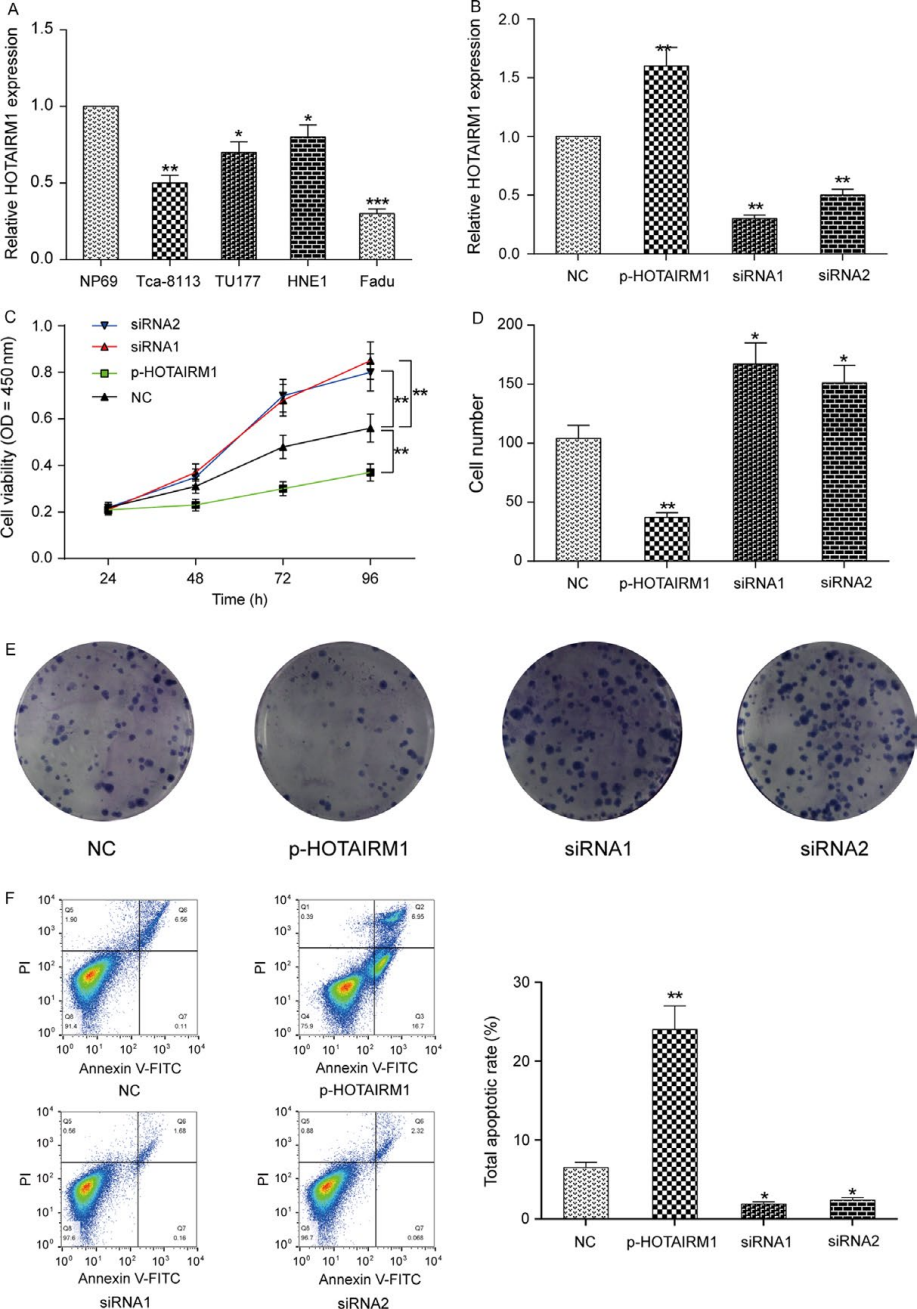
HOTAIRM1 regulated Fadu cell proliferation and apoptosis in vitro (A) QRT‐PCR was used to detect HOTAIRM1 expression levels in different head and neck tumor cells compared with normal cells (NP69 cell). *P <* *0.05, ***P <* *0.001 compared to NP69 cell. (B) HOTAIRM1 expression level was detected in Fadu cells after transfected with pcDNA3.1‐HOTAIR (p‐HOTAIR), siRNA‐HOTAIR (siRNA1 and siRNA2), and no‐load plasmid (negative control, NC) by qRT‐PCR analysis. (C) CCK‐8 assay was used to determine the proliferation of Fadu cells in different transfection groups. (D‐E) Colony formation assay was applied to explore the proliferation of Fadu cells. *P <* *0.05, **P <* *0.01 compared to NC group.

**Figure 3:**
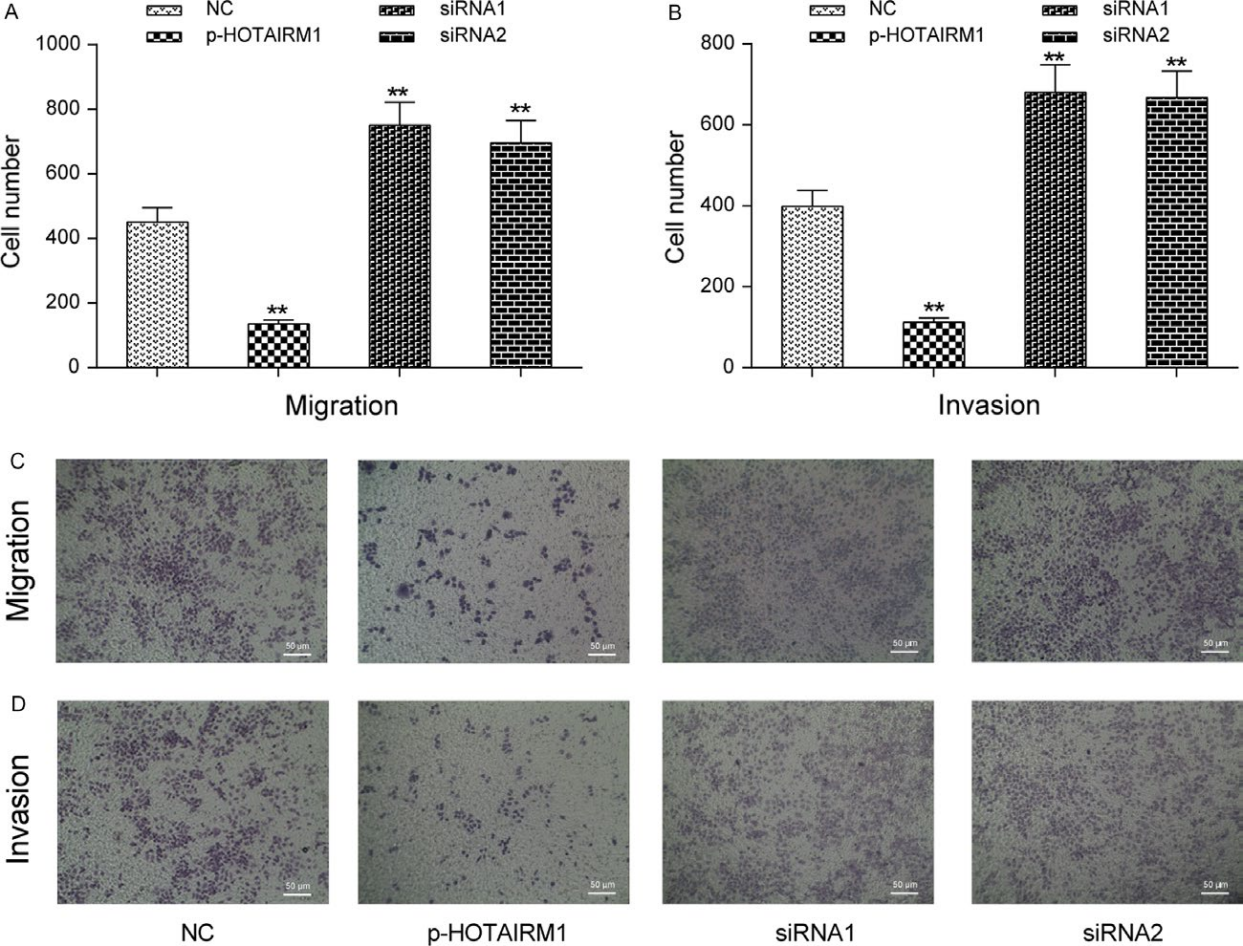
HOTAIRM1 regulated Fadu cell migration and invasion in vitro (A, C) Transwell assay was performed to investigate changes in Fadu cell migration. (B, D) Transwell assay was performed to research changes in Fadu cell invasion. **P <* *0.01 compared to NC group.

First, we tested HOTAIRM1 expression levels in different head and neck tumor cells compared with normal human nasopharyngeal epithelial cells (NP69 cell) and found that HOTAIRM1 expression was the lowest in Fadu cells in four cancer cells (Fig. 2A). So, we selected Fadu cells as our experimental subjects in the following experiments. Next, we investigated the effect of HOTAIRM1 on the proliferation, migration, and invasion of Fadu cells. Fadu cells were assigned to the negative control group (NC, un‐transfected Fadu cells), p‐HOTAIRM1 group (cells transfected with pcDNA3.1‐HOTAIRM1), siRNA1 group (cells transfected with HOTAIRM1 siRNA1), and siRNA1 group (cells transfected with HOTAIRM1 siRNA2). The HOTAIRM1 expression was elevated 70% in the p‐HOTAIRM1 group, whereas the HOTAIRM1 expression significantly decreased in siRNA‐HOTAIRM1 group (Fig. 2B, *P *< 0.01). The CCK‐8 assay showed that HOTAIRM1 overexpression caused a decrease in the cell proliferation of Fadu cells in comparison with NC group (Fig. 2C, *P *< 0.01). Clone formation assay indicated that silencing of HOTAIRM1 promoted the proliferation of Fadu cells compared with NC group (Fig. 2D–E). HOTAIRM1 overexpression observably increased cell apoptosis of Fadu cells (Fig. 2F). HOTAIRM1 overexpression also significantly inhibited migration and invasion in Fadu cells; however, HOTAIRM1 downregulation notably increased migration and invasion in Fadu cells (Fig. 3A–D, *P *< 0.01). Hence, we confirmed that HOTAIRM1 could repress HNT cell growth in vitro.

### HOTAIRM1 suppressed Fadu cell growth in vivo

**Figure 4:**
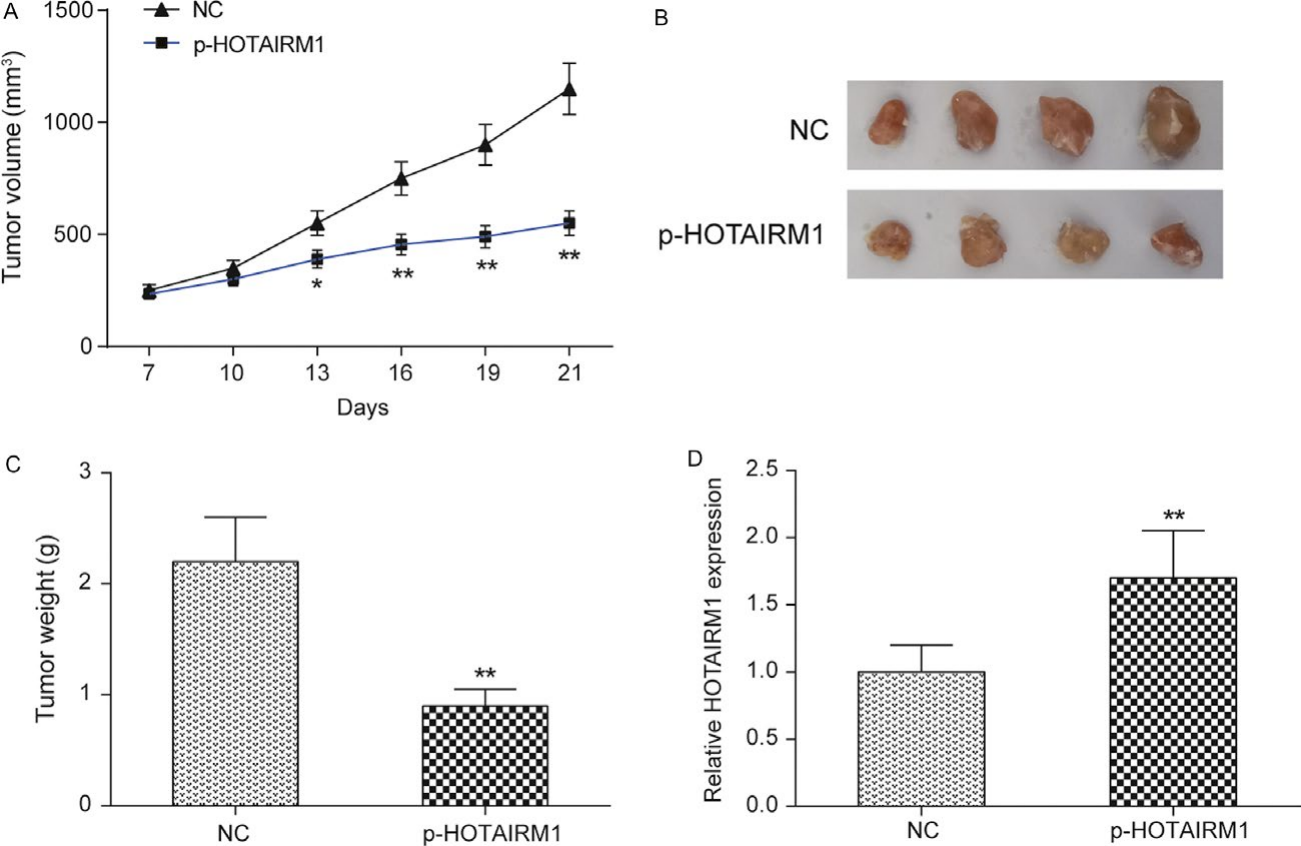
HOTAIRM1 inhibited tumor formation in nude mice (A) After injecting the transfected Fadu cells, the tumor volume was measured for every 3 days. (B) Overexpression of HOTAIRM1 dramatically suppressed HNT tumor growth by comparison to the NC group. (C)Tumor weight was measured at the 21st day after the experiment. (D) The expression level of HOTAIRM1 was significantly higher than NC group in mouse tumor tissue. *P <* *0.05, **P <* *0.01 compared to NC group.

A mouse tumor xenograft model was established to examine the effects of HOTAIRM1 on HNT in vivo. Fadu cells transfected with p‐HOTAIRM1 or NC were injected subcutaneously on the back of each nude mice. After 7 days of the experiment, the tumor volume was measured every three days, and at each point, the Fadu cells with high expression of HOTAIRM1 obviously formed smaller tumor compared to the NC tumor (Fig. 4A). At the termination of the experiment (the 21st day), mice were sacrificed and the tumor was excised (Fig. 4B). The tumor weight in p‐HOTAIRM1 group was significantly less than in NC group (Fig. 4C, *P *< 0.01). Further qRT‐PCR results confirmed the expression of HOTAIRM1 was upregulated 60% in p‐HOTAIRM1 group compared with NC group (Fig. 4D, *P *< 0.01). Taken together, overexpression of HOTAIRM1 efficiently impaired the tumorigenesis ability of Fadu cells in vivo.

### HOTAIRM1 could directly target miR‐148a

**Figure 5:**
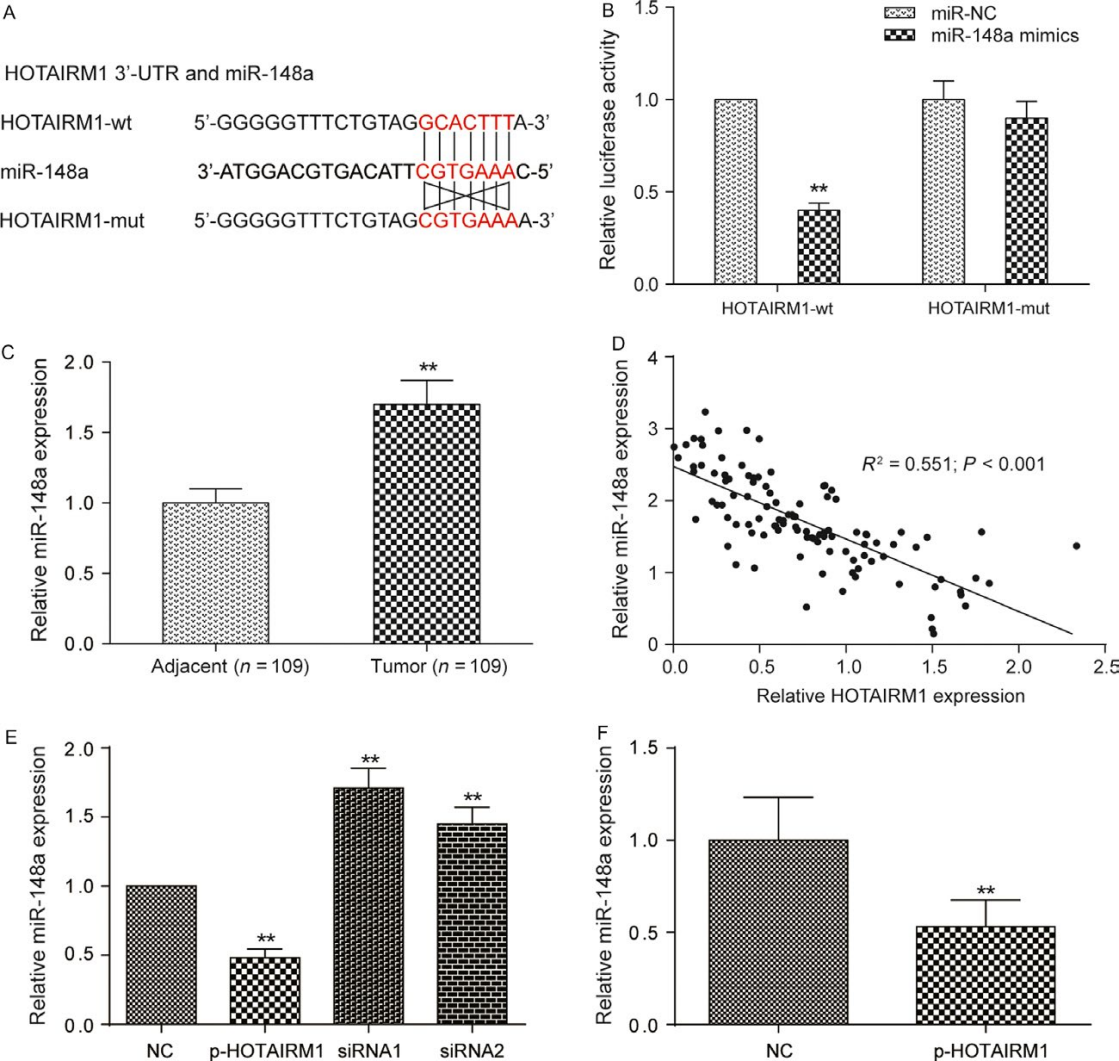
HOTAIRM1 was a target of miR‐148a in Fadu cells (A‐B) Prediction of binding sites of miR‐148a and HOTAIRM1 by bioinformatics (miRcode 11). Then luciferase activity analysis showed transfection of miR‐148a could significantly inhibit the HOTAIRM1 wild‐type luciferase gene carrier fluorescence activity. **P <* *0.01 compared with miR‐NC group. (C) Relative expression of miR‐148a in HNT tumor and normal adjacent tissues. **P <* *0.01 compared with adjacent group. (D) The linear analysis revealed a negative correlation between miR‐148a and HOTAIRM1 expression in HNT tissues (*n *= 109). (E) qRT‐PCR analysis of miR‐148a expression in Fadu cells transfected with p‐NC, p‐HOTAIRM1, HOTAIRM1 siRNA1, and HOTAIRM1 siRNA2. (F) qRT‐PCR analysis of miR‐148a expression in isolated tumor tissues from mice models. **P <* *0.01 compared with miR‐NC group.

We first utilized bioinformatics (miRcode 11) database to predict HOTAIRM1 targets and screen out the miR‐148a which has partial complementary sequence to HOTAIRM1 (Fig. 5A). Then, dual‐luciferase reporter assay was performed to verify the targeted relationship between HOTAIRM1 and miR‐148a in Fadu cells. Results demonstrated that miR‐148a expression was able to significantly suppress the luciferase activity in the miR‐148a mimic and HOTAIRM1‐wt group, but miR‐148a mimic and HOTAIRM1‐mut group showed no obvious difference of luciferase expression compared with miR‐NC in Fadu cells (Fig. 5B, *P *< 0.01). We revealed that miR‐148a expression in tumor tissues was higher for 70% than in adjacent tissues (Fig. 5C, *P *< 0.01). Linear correlation was applied to analyze the relationship between miR‐148a expression and HOTAIRM1 expression in 109 HNT patient samples. A negative correlation was observed as the *R* = −0.7423 and *P *< 0.0001 (Fig. 5D). The p‐HOTAIRM1 group reduced miR‐148a level by about 60% compared NC group in vitro. Increased of HOTAIRM1 expression led to decreased expression levels of miR‐148a and vice versa, indicating that HOTAIRM1 may sponge miR‐148a and impaired its functions (Fig. 5E, *P *< 0.01). In vivo experiments also supported that overexpression of HOTAIRM1 reduced the expression of miR‐148a (Fig. 5F, *P *< 0.01).

### MiR‐148a, a promotor of HNT development, was an inhibitory target of HOTAIRM1

**Figure 6:**
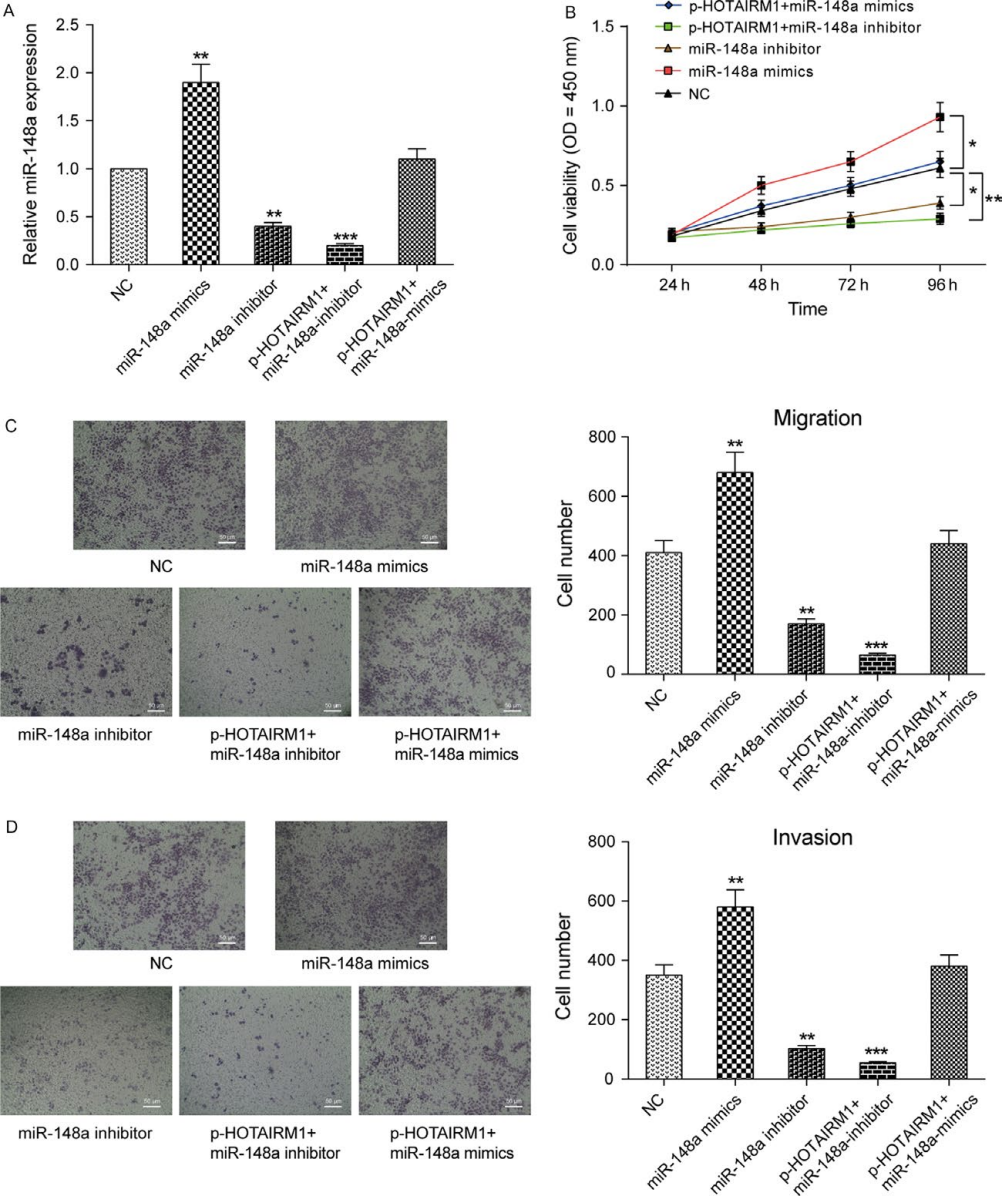
HOTAIRM1 impacted the expression of miR‐148a (A) After Fadu cells were stably transfected with miR‐148a mimics, miR‐148a inhibitor, p‐HOTAIRM1 + miR‐148a inhibitor, or p‐HOTAIRM1 + miR‐148a mimics, the expression of HOTAIRM1 in Fadu cells was analyzed by real‐time PCR. (B) CCK‐8 assay was used to detect cell proliferation in different transfection groups. (C)(D) Transwell assays were performed to investigate changes in Fadu cell migration and invasion. *P <* *0.05, **P <* *0.01, ***P <* *0.001, compared NC group.

Following transfection of Fadu cells with miR‐148a mimics, miR‐148a inhibitor or pcDNA3.1‐HOTAIRM1 respectively, cells were divided into five transfection groups including NC, miR‐148a mimics, miR‐148a inhibitor, p‐HOTAIRM1+miR‐148a inhibitor or p‐HOTAIRM1+miR‐148a mimics group. The expression level of miR‐148a in each group was determined, and the results indicated that the transient transfection was satisfactory. The outcome showed that HOTAIRM1 reduced miR‐148a mimics‐induced expression of miR‐148a (Fig. 6A). MiR‐148a inhibitor group significantly suppressed the proliferation, migration, and invasive abilities of Fadu cells compared with NC group, whereas the miR‐148a mimics group promoted Fadu cells development. The group of p‐HOTAIRM1 + miR‐148a inhibitor was remarkably inhibited the proliferation, migration, and invasive abilities of Fadu cells. The group of p‐HOTAIRM1 + miR‐148a mimics had no great difference with the NC group (Fig. 6B–D).

### DLGAP1 was targeted by miR‐148a and was regulated by HOTAIRM1

**Figure 7:**
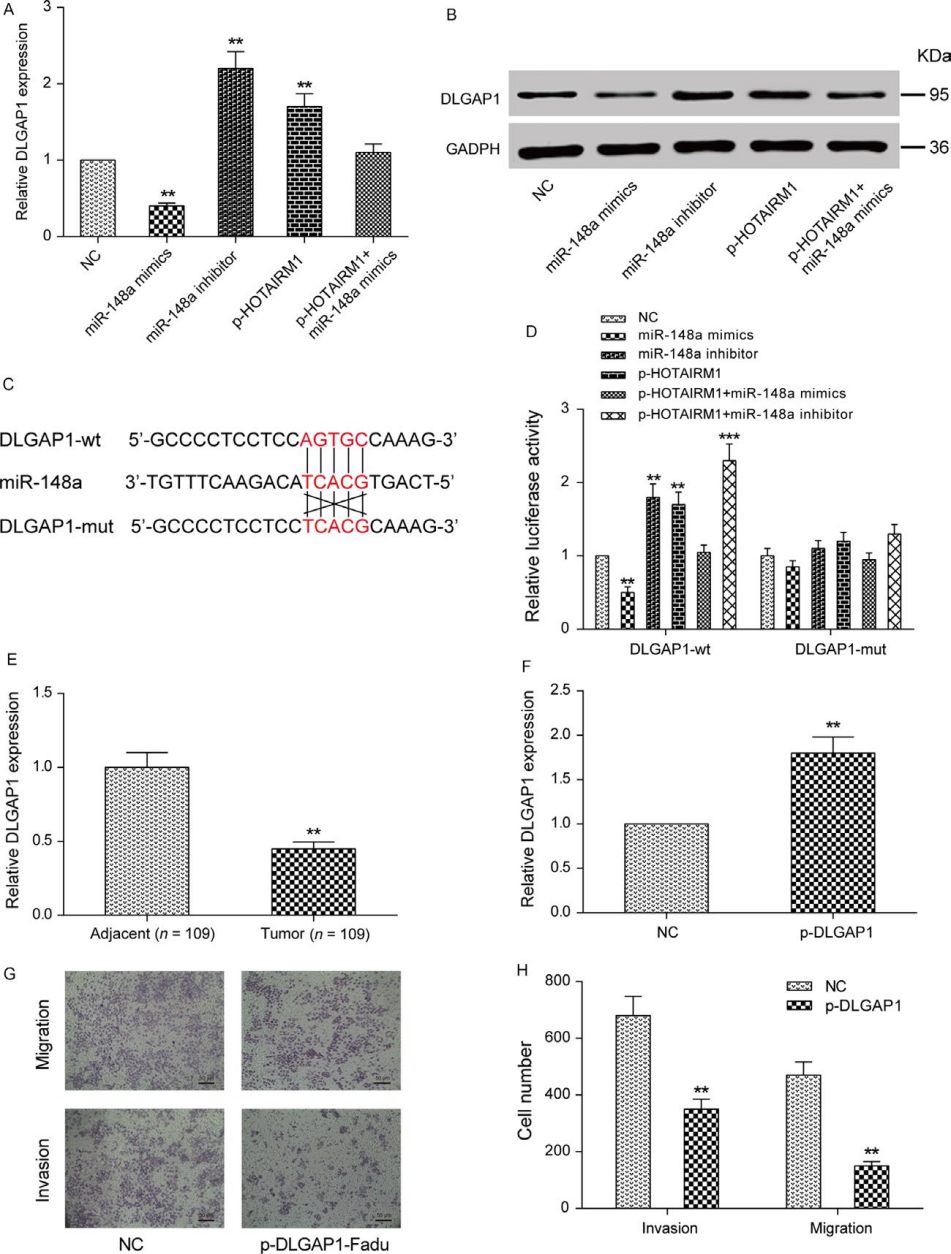
*DLGAP1* was a target protein of miR‐148a and was regulated by HOTAIRM1 (A) qRT‐PCR was performed to determine the relative expression of *DLGAP1* in Fadu cells transfected with miR‐148a mimics, miR‐148a inhibitor, p‐HOTARM1, or p‐HOTAIRM + miR‐148a mimics. (B) Western blot analysis was performed to examine the protein levels of *DLGAP1* in Fadu cells in five transfection group. (C) Prediction of binding sites of miR‐148a and *DLGAP1* by bioinformatics (RNA22 v2.0). (D) Luciferase activity analysis, transfection of miR‐148a could significantly inhibit the *DLGAP1* wild‐type luciferase gene carrier fluorescence activity. (E) *DLGAP1* expression level was determined in tumor tissues and adjacent tissues using qRT‐PCR. **P <* *0.01 compared with adjacent group. (F) *DLGAP1* expression level was detected in Fadu cells after transfected with pcDNA3.1‐*DLGAP1* (p‐*DLGAP1*) and no‐load plasmid (NC) by qRT‐PCR analysis. (G‐H) Overexpression of *DLGAP1* suppressed Fadu cell migration and invasion. **P <* *0.01, ***P <* *0.001 compared with the NC group.

Bioinformatics analysis (RNA22 v2.0) was used to predict target genes for miR‐148a and found *DLGAP1* as one of the best candidates. Given the findings that *DLGAP1* was a target of miR‐148a, we sought to determine whether the regulation of *DLGAP1* expression by HOTAIRM1 was dependent on miR‐148a. As expected, evaluated miR‐148a expression inhibited the mRNA expression of *DLGAP1,* while the expression level of *DLGAP1* increased after downregulation of miR‐148a. However, there were no apparent changes of qRT‐PCR and Western blot results in p‐HOTARM1 + miR‐148a mimics group compared with NC group (Fig. 7A–B, *P *< 0.01). Base pairing between miR‐148a and the putative target site in the *DLGAP1*‐wt was predicted, and the *DLGAP1*‐mut 3′UTR was constructed according to bioinformatics analysis (Fig. 7C). Dual‐luciferase activity assay suggested that miR‐148a mimics reduced the luciferase activity of *DLGAP1*‐wt, while miR‐148a inhibitor, p‐HOTAIRM1, and p‐HOTAIRM1 + miR‐148a inhibitor significantly increased the luciferase activity of *DLGAP1*‐wt. The p‐HOTAIRM1 + miR‐148a mimics restored the luciferase activity of *DLGAP1*‐wt to control level. But all transfection groups could not affect the luciferase activity of *DLGAP1*‐mut (Fig. 7D). The *DLGAP1* expressions in tumor tissues were downregulated for about 50% compared with adjacent normal tissues (Fig. 7E, *P *< 0.01). Next, we investigated the effect of *DLGAP1* on the migration and invasion of Fadu cells. The experimental group (p‐*DLGAP1* group) of Fadu cells was transfected with pcDNA3.1‐*DLGAP1*, and the control group (NC group) of Fadu cells was transfected with pcDNA3.1 vector. Overexpression of *DLGAP1* enhanced the expression levels of *DLGAP1* for about 80% compared with NC group (Fig. 7F, *P *< 0.01). Transwell assay revealed that overexpressing *DLGAP1* significantly inhibited migration and invasion of Fadu cell (Fig. 7G–H, *P *< 0.01).

## Discussion

In the HNT tissues, expression of HOTAIRM1 was significantly downregulated compared with the adjacent normal tissues, indicating that HOTAIRM1 may act as an anti‐oncogene in HNT. We found that overexpression of HOTAIRM1 inhibited Fadu cell proliferation, attenuated cell migration, and invasion in vitro. Mouse tumor xenograft models were established which confirmed that overexpression of HOTAIRM1 suppressed tumor growth in vivo. Moreover, an obvious inverse correlation between miR‐148a and HOTAIRM1 in Fadu cell was observed, and upregulation of miR‐148a was found to increase the growth, metastasis, and invasion of Fadu cell. *DLGAP1* was further identified as a direct target of miR‐148a. The expression level of *DLGAP1* was significantly downregulated by overexpression of miR‐148 in Fadu cell, while upregulation of *DLGAP1* plays a tumor‐suppressing role in Fadu cells.

HOTAIRM1 has been reported to play a significant role in many types of tumor. Recent study indicated that HOTAIRM1 is a tumor suppressor by affecting a series of genes related to cell proliferation in acute myeloid leukemia 7. Relying on the two PU.1 motifs at the *HOTAIRM1* promoter, the increasing expression of PU.1 contributes to the dysregulation of HOTAIRM1 in acute myeloid leukemia cells 8. Wan et al. 9 also found that expression of HOTAIRM1 was decreased in colorectal cancer tissues compared with matched normal tissues. Same as their observation, we found that the level of HOTAIRM1 in HNT tissues was significantly less than in adjacent normal tissues. Up to date, the effects and mechanisms of HOTAIRM1 on HNT tumorigenesis and progression are still largely unclear. Therefore, we researched the expression and function of HOTAIRM1 in HNT. After overexpression of HOTAIRM1, cell proliferation, migration, and invasion were significantly inhibited in vitro as well as the tumor growth in vivo. These results suggest that HOTAIRM1 contributes to oncogenesis inhibition of HNT.

Recent researches illuminated that lncRNAs could act as an endogenous miRNA sponges and involve in posttranscriptional regulation by interaction with miRNAs 21, 22. Numerous of lncRNAs have been included in, including lncRNA CCAT1 21 and NEAT1 23. For example, Li et al. reported that lncRNA H19 could act as a sponge of miR‐630 to control the enhancer of zeste homolog 2 (EZH2) expressions in nasopharyngeal carcinoma. In this study, we identified interaction between HOTAIRM1 and miR‐148a by sequence complementarity analysis and dual‐luciferase reporter assay. In the former research, miR‐148a has different functions in numerous cancers 17. Li et al. 17 revealed that miR‐148a may serve as a tumor suppressor by negatively regulating nasopharyngeal carcinoma. In contrast, miR‐148a has also been reported to be able to enhance the progress of multiple kinds of cancers, including osteosarcoma 16, glioblastoma 24, and gastric cancer 25. Similarly, we found that miR‐148a mimics promoted cell ability in Fadu cell, whereas miR‐148a inhibitor revealed a converse effect. Importantly, upregulation of miR‐148a almost reversed the phenotype induced by HOTAIRM1 overexpression. These results indicated that miR‐148a is downstream of HOTAIRM1 in a signaling cascade that regulated HNT progression. Based on the sequence complementarity between HOTAIRM1 and miR‐148a, we believe that miR‐148a is likely a direct target of HOTAIRM1. Many other factors affected RNA molecules interaction with lncRNA; we therefore could not exclude the possibility that HOTAIRM1 targeted other miRNAs. And it may serve as an intermediate regulator of the expression of miR‐148a.

For further understanding, the regulatory mechanism of miR‐148a in HNT progression, the potential targeted genes, and the related pathways were taken into consideration. *DLGAP1* was predicted as a direct target of miR‐148a at its 3′‐UTR mRNA by bioinformatics. Although the effect of *DLGAP1* in HNT has not been clearly studied, it has been proved that DLGAP protein family was linked to their function in the brain and involvement in neurological diseases 26. In the present study, we revealed that miR‐124 regulates HNT growth by targeting *DLGAP1*. Given that *DLGAP1* interacts with miR‐148a in Fadu cell lines, we concluded that HOTAIRM1 may regulate *DLGAP1* through miR‐148a.

Our study also has several limitations. First, there could be other miRNAs sponged by HOTAIRM1 and contributing to the progression and metastasis of HNT, as well as other gene targets of miR‐148a. Moreover, the mechanisms for how HOTAIRM1 is transcriptionally regulated in HNT were not deeply investigated in this manuscript.

## Conclusion

In summary, we identified that increased HOTAIRM1 expression was capable to significantly suppress HNT cell proliferation, migration, invasion in vitro and inhibited tumorigenesis in vivo. Moreover, sufficient evidences indicated that the antitumor effect of HOTAIRM1 was generated via sponging miR‐148a and thus facilitating the expression of *DLGAP1*, which provided three molecular targets for therapeutic intervention of HNT.

## Conflict of Interest

There are no conflict of interests or financial ties to disclose from any authors.

## Research Involving Human Participants or Animals

This study involved human participants or animals and was approved by the School and Hospital of Stomatology, Shandong University.

## Informed Consent

All the patients and participants have signed the informed consent.
